# Prophylactic treatment of dacomitinib‐induced skin toxicities in epidermal growth factor receptor‐mutated non–small‐cell lung cancer: A multicenter, Phase II trial

**DOI:** 10.1002/cam4.6184

**Published:** 2023-06-03

**Authors:** Masahiro Iwasaku, Junji Uchino, Kenji Chibana, Shigeru Tanzawa, Takahiro Yamada, Kazunori Tobino, Yasuki Uchida, Takashi Kijima, Katsumi Nakatomi, Miiru Izumi, Nobuyo Tamiya, Hideharu Kimura, Masaki Fujita, Ryoichi Honda, Chieko Takumi, Tadaaki Yamada, Yoshiko Kaneko, Fumiaki Kiyomi, Koichi Takayama

**Affiliations:** ^1^ Department of Pulmonary Medicine, Graduate School of Medical Science Kyoto Prefectural University of Medicine Kyoto Japan; ^2^ Department of Respiratory Medicine National Hospital Organization Okinawa National Hospital Okinawa Japan; ^3^ Division of Medical Oncology, Department of Internal Medicine Teikyo University School of Medicine Tokyo Japan; ^4^ Department of Pulmonary Medicine Matsushita Memorial Hospital Osaka Japan; ^5^ Department of Respiratory Medicine Iizuka Hospital Iizuka Japan; ^6^ Division of Respiratory Medicine, Department of Internal Medicine Shiga University of Medical Science Japan; ^7^ Department of Respiratory Medicine and Hematology Hyogo Medical University, School of Medicine Hyogo Japan; ^8^ Department of Respiratory Medicine National Hospital Organization Ureshino Medical Center Ureshino Japan; ^9^ Department of Respiratory Medicine National Hospital Organization, Omuta National Hospital Fukuoka Japan; ^10^ Department of Pulmonary Medicine Rakuwakai Otowa Hospital Kyoto Japan; ^11^ Department of Respiratory Medicine Kanazawa University Hospital Ishikawa Japan; ^12^ Department of Respiratory Medicine Fukuoka University Hospital Fukuoka Japan; ^13^ Department of Respiratory Medicine Asahi General Hospital Asahi Japan; ^14^ Department of Respiratory Medicine Japanese Red Cross Kyoto Daiichi Hospital Kyoto Japan; ^15^ Statistics and Data Center, Clinical Research Support Center Kyushu Fukuoka Japan

**Keywords:** dacomitinib, dermatitis, non‐small‐cell lung cancer, steroids, sunscreen

## Abstract

**Background:**

Dacomitinib significantly improves progression‐free survival and overall survival (OS) compared with gefitinib in patients with non–small‐cell lung cancer (NSCLC) harboring epidermal growth factor receptor (EGFR)‐activating mutations. However, dacomitinib often causes skin toxicities, resulting in treatment discontinuation. We aimed to evaluate a prophylactic strategy for skin toxicity induced by dacomitinib.

**Methods:**

We performed a single‐arm, prospective, open‐label, multi‐institutional phase II trial for comprehensive skin toxicity prophylaxis. Patients with NSCLC harboring EGFR‐activating mutations were enrolled and received dacomitinib with comprehensive prophylaxis. The primary endpoint was the incidence of skin toxicity (Grade ≥2) in the initial 8 weeks.

**Results:**

In total, 41 Japanese patients participated between May 2019 and April 2021 from 14 institutions (median age 70 years; range: 32–83 years), 20 were male, and 36 had a performance status of 0–1. Nineteen patients had exon 19 deletions and L858R mutation. More than 90% of patients were perfectly compliant with prophylactic minocycline administration. Skin toxicities (Grade ≥2) occurred in 43.9% of patients (90% confidence interval [CI], 31.2%–56.7%). The most frequent skin toxicity was acneiform rash in 11 patients (26.8%), followed by paronychia in five patients (12.2%). Due to skin toxicities, eight patients (19.5%) received reduced doses of dacomitinib. The median progression‐free survival was 6.8 months (95% CI, 4.0–8.6 months) and median OS was 21.6 months (95% CI, 17.0 months–not reached).

**Conclusion:**

Although the prophylactic strategy was ineffective, the adherence to prophylactic medication was quite good. Patient education regarding prophylaxis is important and can lead to improved treatment continuity.

## Graphical abstract

We evaluated whether comprehensive prophylaxis reduces dacomitinib‐induced skin toxicity and improves treatment continuation. More than 90% of patients were perfectly compliant with prophylactic minocycline administration.

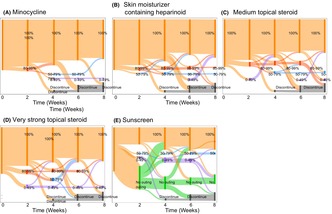

## 1 INTRODUCTION

The current standard of care for patients with advanced or metastatic non–small‐cell lung cancer (NSCLC) harboring epidermal growth factor receptor (EGFR)‐activating mutations is treatment with EGFR‐tyrosine kinase inhibitors (TKIs). A meta‐analysis of randomized controlled trials with first‐generation EGFR‐TKIs (gefitinib or erlotinib) showed prolonged progression‐free survival (PFS) relative to chemotherapy in first‐line settings.^1^ Dacomitinib, a second‐generation EGFR‐TKI, has demonstrated significantly improved PFS and overall survival (OS) compared with gefitinib (PFS, 14.7 vs. 9.2 months; OS, 34.1 vs. 26.8 months).^2^, ^3^ However, dacomitinib results in more side effects than gefitinib. Dacomitinib is an irreversible TKI and differs from gefitinib, a reversible TKI, which may also be related to its toxicity.^4^ Dacomitinib also differs from other EGFR‐TKIs in its metabolic pathway since it is mediated by cytochrome P450 (CYP) 2D6 and CYP2A4.^5^ As for toxicity, skin and mucous membrane toxicities, such as oral mucositis, paronychia, and diarrhea, have been found to be more frequent and severe with dacomitinib use than with gefitinib use. Such skin toxicity is troublesome and the primary reason for discontinuing dacomitinib treatment.^2^ Therefore, managing skin toxicity is essential for its continuation.

In the ARCHER1042 study, preemptive treatment with doxycycline reduced skin toxicity of dacomitinib treatment.^6^ A systematic review also showed a prophylactic effect of tetracyclines on skin damage associated with EGFR inhibitors.^7^ In addition to oral administration of antibiotics, skin and sun protection using skin moisturizers, topical steroids, and sunscreen are also expected to be effective. These topical agents, as single agents, have not prevented chemotherapy‐induced skin damage significantly. However, topical steroids are recommended for treatment of acneiform rash by EGFR‐TKIs.^8^ Additionally, prophylactic use of skin moisturizers prevented skin dryness induced by EGFR‐TKIs.^9^ Furthermore, combined prophylactic treatments (oral minocycline, topical steroid, skin moisturizer, and sunscreen), in a comprehensive prophylactic strategy, prevent skin toxicity caused by panitumumab, which is an antibody agent targeting EGFR.^10^ Comprehensive skin toxicity prophylaxis may also reduce dacomitinib‐induced skin toxicity and improve treatment continuation; however, this hypothesis has not yet been tested. Therefore, we aimed to evaluate the ability of prophylactic treatment to reduce skin toxicity induced by dacomitinib and ensure treatment continuity.

## 2 METHODS

### 2.1 Study design and patient selection

This multi‐institutional phase II trial employed a single‐arm, prospective, and open‐label design. The study protocol has been previously reported and registered in the Japan Registry of Clinical Trials (jRCTs071190015, Figure S1).^11^

The key eligibility criteria were as follows: patients with advanced or recurrent NSCLC excluding squamous cell carcinoma, harboring EGFR‐activating mutations, aged ≥20 years, a performance status (PS) of ≤2 based on the Eastern Cooperative Oncology Group scale, and adequate organ function. The main exclusion criteria were symptomatic brain metastases and interstitial lung disease.

### 2.2 Treatments

Patients can participate in the study regardless of prior treatment for NSCLC. If the patients have received prior therapy, the following periods must have elapsed before study entry and adverse events: at least 2 weeks after cytotoxic chemotherapy administration and 4 weeks after chest irradiation. If the patient received EGFR‐TKI treatment, skin toxicities must have recovered to Grade <2 based on the National Cancer Institute Common Terminology Criteria for Adverse Events (CTCAE) scale for eligibility. All adverse events were graded according to CTCAE, version 5.0.

Eligible patients received dacomitinib and comprehensive prophylaxis for skin toxicity. Dacomitinib was started at 45 mg/day and tolerated for up to two dose reductions (30 and 15 mg/day). The preemptive strategy consisted of five agents: minocycline (100 mg orally, once daily); skin moisturizer containing heparinoid (twice daily for the face, neck, chest, back, and extremities); a medium topical steroid (class IV, twice daily for the face); a very strong topical steroid (class II, twice daily for the neck, chest, back, and extremities); and sunscreen (sun protection factor ≥ 25, protection factor UVA 4–8, and UVA and UVB protection). We provided patient guidance for medicine use and evaluated their adherence to the prophylactic treatment using a patient diary.

Treatments were discontinued when the following events occurred: (1) disease progression; (2) severe adverse events (interstitial pneumonitis of any grade or non‐hematological toxicities of Grade 4); (3) requirement for threefold dose reduction of dacomitinib administration; and (4) discontinuation of dacomitinib treatment for ≥3 weeks. Severe adverse events led to the interruption of dacomitinib treatment, which could restart after recovery. In the case of diarrhea or skin toxicities, dacomitinib administration was restarted after improvement to Grade ≤1. In the case of other adverse events, dacomitinib was restarted after recovery with Grade ≤2.

### 2.3 Endpoints

The primary endpoint was the incidence of skin toxicities ranked as Grade ≥2 during the initial 8 weeks of dacomitinib treatment. Dermatologic toxicity was defined as acneiform rash, dry skin, pruritus, paronychia, palmar‐plantar erythrondysesthesia syndrome, maculopapular rash, erythema multiforme, skin infection, and eczema. Secondary endpoints included the incidence of dose reduction of dacomitinib, PFS, and safety.

### 2.4 Statistical analysis

We planned to include 40 patients with NSCLC in this trial. The sample size was calculated using a one‐sample binomial test. In the ARCHER1042 trial, the incidence of Grade ≥2 skin toxicity during 8 weeks of dacomitinib initiation was 46% in the placebo group and 23% in the doxycycline prophylaxis group, respectively.^4^ Assuming a conservative skin toxicity incidence of 23% in this study, 36 patients were required with a threshold of 46%, one‐sided significance level of 5%, and power of 90%. Assuming a discontinuation rate of 10%, a planned sample size of 40 was determined.

Regarding the primary endpoint, the incidence of Grade ≥2 skin toxicities in the initial 8 weeks of dacomitinib treatment was calculated, and a 90% confidence interval (CI) was estimated based on the Wald method. The 95% CI was calculated for the incidence of dose reduction with dacomitinib. The survival curve of PFS was estimated using the Kaplan–Meier method, and the 95% CI of the median PFS and yearly PFS rates were estimated using the Brookmeyer and Crowley method and Greenwood method, respectively. SAS version 9.4 (SAS Institute, Cary, NC, USA) was used for all statistical analyses.

### 2.5 Ethics considerations

This study was conducted in accordance with the principles of the Declaration of Helsinki. The study protocol and all amendments were reviewed and approved by the ethical review board of the Clinical Research Network Fukuoka Certified Review Board (CRB7180004). All patients provided written informed consent before enrollment.

## 3 RESULTS

### 3.1 Patients

A total of 41 patients with EGFR‐mutated NSCLC were enrolled between May 2019 and April 2021 from 14 institutions. The data cut‐off date was April 30, 2022. The baseline patient characteristics are summarized in Table 1. Among the 41 enrolled patients, the median age was 70 years (range: 32–83 years), 20 (48.8%) were male, and 36 (87.8%) had a PS of 0–1. Regarding EGFR mutation status, 19 (46.3%) patients had exon 19 deletions and L858R mutation. Moreover, 18 (43.9%) patients were current or former smokers. A total of 31 patients (75.6%) had metastasis, with 14 (34.1%) in the brain, 13 (31.7%) in the bones, and 7 (17.1%) in the liver. Furthermore, seven patients (17.1%) received dacomitinib therapy as first‐line treatment and 34 (82.9%) had received previous anticancer therapy with a median of two regimens. Previous therapies included EGFR‐TKIs (26 patients [63.4%]), anticancer agents other than EGFR‐TKIs (21 patients [51.2%]), and surgical resection (15 patients [36.6%]) (Table 1).

**TABLE 1:** Baseline patient characteristics (*N*=41).

Variables	N (%)
Age (years), median (range)	70 (32‐83)
Male	20 (48.8)
Height (cm), median (range)	158.3 (137.0‐174.0)
Weight (kg), median (range)	57.6 (36.4‐80.9)
Body surface^a^ (m^2^), mean (SD)	1.56 (0.20)
Current or former smoker	18 (43.9)
Performance Status	
0	16 (39.0)
1	20 (48.8)
2	5 (12.2)
Histology	
Adenocarcinoma	40 (97.6)
LCNEC	1 (2.4)
EGFR mutation status	
Exon 19 del	19 (46.3)
L858R	19 (46.3)
Others^b^	3 (7.3)
Stage	
IIIB, IIIC	2 (4.9)
IV	28 (68.3)
Postoperative recurrence	11 (26.8)
Metastatic organs	
Brain	14 (34.1)
Bone	13 (31.7)
Liver	7 (17.1)
Adrenal gland	5 (12.2)
Previous treatments	
EGFR‐TKIs	26 (63.4)
Chemotherapy	21 (51.2)
Surgical resection	15 (36.6)
Radiation	11 (26.8)
Number of previous treatments	
0	7 (17.1)
1	10 (24.4)
2	6 (14.6)
3‐	18 (43.9)

Abbreviations: EGFR, epidermal growth factor receptor; LCNEC, large‐cell neuroendocrine carcinoma; TKIs, tyrosine kinase inhibitors.

^a^ Body surface area was calculated using the Du Bois formula.

^b^ One patient each had L861Q, exon20 insertion, and G719X compounded with S768I.

### 3.2 Medication adherence

**FIGURE 1:**
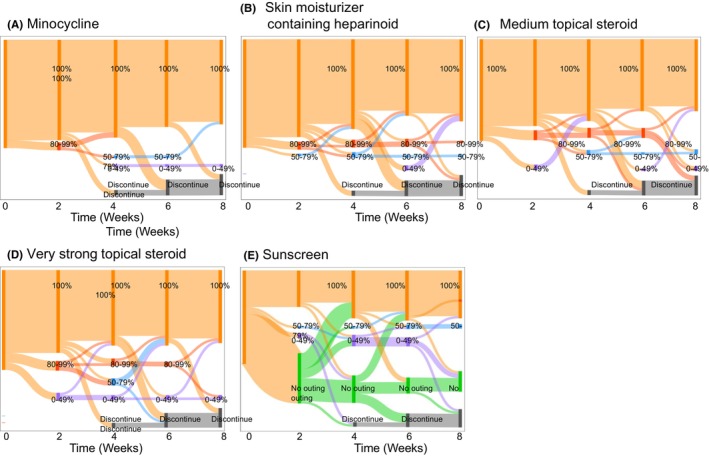
Adherence pattern for prophylaxis medication during the 8 weeks of the study period. Adherence to prophylaxis medication was evaluated every 2 weeks for 8 weeks. The respective numbers of patients who were 100% compliant with prophylaxis at weeks 2, 4, 6, and 8 with each treatment were as follows: (A) minocycline: 38, 37, 33, and 32; (B) skin moisturizer: 37, 34, 29, and 31; (C) medium topical steroid: 35, 33, 29, and 29; (D) very strong topical steroid: 34, 31, 30, and 30; (E) sunscreen: 16, 21, 22, and 21. Eight patients discontinued dacomitinib treatments before the end of week 8.

Of the 41 enrolled patients, more than 90% were perfectly compliant with prophylactic minocycline administration throughout the study period. Adherence to the topical medications was lower than that to the oral prophylaxis; nevertheless, more than 80% of the patients were compliant with the topical medications (skin moisturizer containing heparinoid and topical steroids) in 80% of the days during the study period. Sunscreen was recommended for use on outings, and perfect adherence was observed throughout >70% of the period (Figure 1).

### 3.3 Primary endpoint

Skin toxicities (Grade ≥2) occurred in 43.9% (90% CI, 31.2%–56.7%) of the enrolled patients during the initial 8 weeks of dacomitinib therapy, which did not meet the primary endpoint (*p* = 0.394). The most frequent skin toxicity of Grade ≥2 was acneiform rash in 11 patients (26.8%), followed by paronychia in 5 patients (12.2%) (Table 2). A total of 39 patients (95.1%) had skin toxicity of any grade. Paronychia was the most common (31 patients, 75.6%), followed by acneiform rash (28 patients, 68.3%). No significant correlation was observed between skin toxicity (Grade ≥2) and patient characteristics (Table S1).

**TABLE 2:** Skin toxicities in the initial 8 weeks of treatment (*n* = 41).

	Grade ≥2	Any Grade	Grade 1	Grade 2	Grade 3	Grade 4
All events, n (%)	18 (43.9)	39 (95.1)	21 (51.2)	14 (34.1)	4 (9.8)	‐
Acneiform rash	11 (26.8)	28 (68.3)	17 (41.5)	10 (24.4)	1 (2.4)	‐
Paronychia	5 (12.2)	31 (75.6)	26 (63.4)	4 (9.8)	1 (2.4)	‐
Pruritus	3 (7.3)	14 (34.1)	11 (26.8)	3 (7.3)	‐	‐
Rash macro‐popular	3 (7.3)	14 (34.1)	11 (26.8)	3 (7.3)	‐	‐
Erythema multiforme	3 (7.3)	6 (14.6)	3 (7.3)	1 (2.4)	2 (4.9)	‐
Dry skin	2 (4.9)	28 (68.3)	26 (63.4)	2 (4.9)	‐	‐
Palmar‐plantar erythrodysesthesia syndrome	1 (2.4)	4 (9.8)	3 (7.3)	1 (2.4)	‐	‐
Skin infection	1 (2.4)	2 (4.9)	1 (2.4)	1 (2.4)	‐	‐
Eczema	‐	2 (4.9)	2 (4.9)	‐	‐	‐

### 3.4 Dacomitinib treatment in the initial 8 weeks

During the 8 weeks after the start of dacomitinib therapy, nine patients (22.2%) discontinued treatment with dacomitinib. Disease progression was the most common cause (five patients). Among the patients who discontinued dacomitinib within the 8 weeks, the median treatment duration was 22 days (range: 8–52 days) (Table S2). Interruption of dacomitinib therapy during the 8 weeks was observed in 21 patients (51.2%). Of the 21 patients, 5 were underwent repeated interruption (Table S3).

### 3.5 Dose modification and safety

Due to skin toxicities, eight patients (19.5%; 95% CI, 7.4%–31.6%) underwent dose reduction. Of the eight patients, seven reduced the dacomitinib dosage once and one reduced the dose twice. The reasons for dose reduction mainly included acneiform rash (six patients), maculopapular rash (three patients), and paronychia (two patients).

In addition to skin toxicities, severe non‐hematological adverse events (Grade ≥3) occurred in 12 patients (29.3%) (Table 3). Oral mucositis was the most frequent adverse event (four patients, 9.8%), followed by anorexia (three patients, 7.3%). Grade 3 interstitial lung disease occurred in one patient, while Grade 3 diarrhea was reported in another one patient.

**TABLE 3:** Adverse events other than skin toxicities in the initial 8 weeks of treatment (*n*=41).

Adverse event	Grade ≥3	Any Grade	Grade 1	Grade 2	Grade 3	Grade 4
Non‐hematological adverse events, *n* (%)						
All events (Non‐hematological)	12 (29.3)	40 (97.6)	11 (26.8)	17 (41.5)	11 (26.8)	1 (2.4)
Oral mucositis	4 (9.8)	25 (61.0)	13 (31.7)	8 (19.5)	4 (9.8)	‐
Anorexia	3 (7.3)	18 (43.9)	11 (26.8)	4 (9.8)	3 (7.3)	‐
Diarrhea	1 (2.4)	34 (82.9)	22 (53.7)	11 (26.8)	1 (2.4)	‐
Fatigue	1 (2.4)	18 (43.9)	12 (29.3)	5 (12.2)	1 (2.4)	‐
Nausea	1 (2.4)	10 (24.4)	8 (19.5)	1 (2.4)	1 (2.4)	‐
Vomiting	1 (2.4)	3 (7.3)	1 (2.4)	1 (2.4)	1 (2.4)	‐
Musculoskeletal and connective tissue disorder‐other, specify	1 (2.4)	1 (2.4)	‐	‐	‐	1 (2.4)
Pneumonitis	1 (2.4)	1 (2.4)	‐	‐	1 (2.4)	‐
Seizure	1 (2.4)	1 (2.4)	‐	‐	1 (2.4)	‐
Anemia	‐	20 (48.8)	15 (36.6)	5 (12.2)	‐	‐
Alopecia	‐	2 (4.9)	1 (2.4)	1 (2.4)	‐	‐
Arthralgia	‐	2 (4.9)	1 (2.4)	1 (2.4)	‐	‐
Malaise	‐	2 (4.9)	1 (2.4)	1 (2.4)	‐	‐
Edema in the limbs	‐	2 (4.9)	2 (4.9)	‐	‐	‐
Myalgia	‐	2 (4.9)	2 (4.9)	‐	‐	‐
Upper respiratory infection	‐	1 (2.4)	‐	1 (2.4)	‐	‐
Tremor	‐	1 (2.4)	‐	1 (2.4)	‐	‐
Lung infection	‐	1 (2.4)	‐	1 (2.4)	‐	‐
Abdominal pain	‐	1 (2.4)	‐	1 (2.4)	‐	‐
Peripheral sensory neuropathy	‐	1 (2.4)	‐	1 (2.4)	‐	‐
Dysesthesia	‐	1 (2.4)	‐	1 (2.4)	‐	‐
Blurred vision	‐	1 (2.4)	‐	1 (2.4)	‐	‐
Pain	‐	1 (2.4)	‐	1 (2.4)	‐	‐
Dry eye	‐	1 (2.4)	1 (2.4)	‐	‐	‐
Cheilitis	‐	1 (2.4)	1 (2.4)	‐	‐	‐
Pain in extremity	‐	1 (2.4)	1 (2.4)	‐	‐	‐
Atrial fibrillation	‐	1 (2.4)	1 (2.4)	‐	‐	‐
Renal dysfunction	‐	1 (2.4)	1 (2.4)	‐	‐	‐
Renal calculi	‐	1 (2.4)	1 (2.4)	‐	‐	‐
Headache	‐	1 (2.4)	1 (2.4)	‐	‐	‐
Dizziness	‐	1 (2.4)	1 (2.4)	‐	‐	‐
Constipation	‐	1 (2.4)	1 (2.4)	‐	‐	‐
Hematological adverse events						
Hypoalbuminemia^a^	1 (2.5)	36 (90.0)	28 (70.0)	7 (17.5)	1 (2.5)	‐
Hypokalemia	1 (2.4)	13 (31.7)	12 (29.3)	‐	‐	1 (2.4)
Platelet count decreased	1 (2.4)	8 (19.5)	7 (17.1)	‐	‐	1 (2.4)
Hyperuricemia	1 (2.4)	1 (2.4)	‐	‐	1 (2.4)	‐
Anemia	‐	20 (48.8)	15 (36.6)	5 (12.2)	‐	‐
Creatinine increased	‐	15 (36.6)	12 (29.3)	3 (7.3)	‐	‐
Alkaline phosphatase increased	‐	14 (34.1)	12 (29.3)	2 (4.9)	‐	‐
Hyponatremia	‐	13 (31.7)	12 (29.3)	1 (2.4)	‐	‐
Aspartate aminotransferase increased	‐	12 (29.3)	11 (26.8)	1 (2.4)	‐	‐
Hypocalcemia^b^	‐	11 (29.7)	8 (21.6)	3 (8.1)	‐	‐
Alanine aminotransferase increased	‐	11 (26.8)	10 (24.4)	1 (2.4)	‐	‐
Hyperkalemia	‐	6 (14.6)	5 (12.2)	1 (2.4)	‐	‐
White blood cell decreased	‐	4 (9.8)	1 (2.4)	3 (7.3)	‐	‐
Neutrophil cell count decreased	‐	4 (9.8)	3 (7.3)	1 (2.4)	‐	‐
Hypernatremia	‐	3 (7.3)	3 (7.3)	‐	‐	‐
Hypercalcemia†	‐	2 (5.4)	2 (5.4)	‐	‐	‐
Hemoglobin increased	‐	1 (2.4)	1 (2.4)	‐	‐	‐
Total bilirubin increased	‐	1 (2.4)	1 (2.4)	‐	‐	‐

^a^ Number of evaluated population = 40 patients.

^b^ Number of evaluated population = 37 patients.

### 3.6 Efficacy and long‐term safety

**FIGURE 2:**
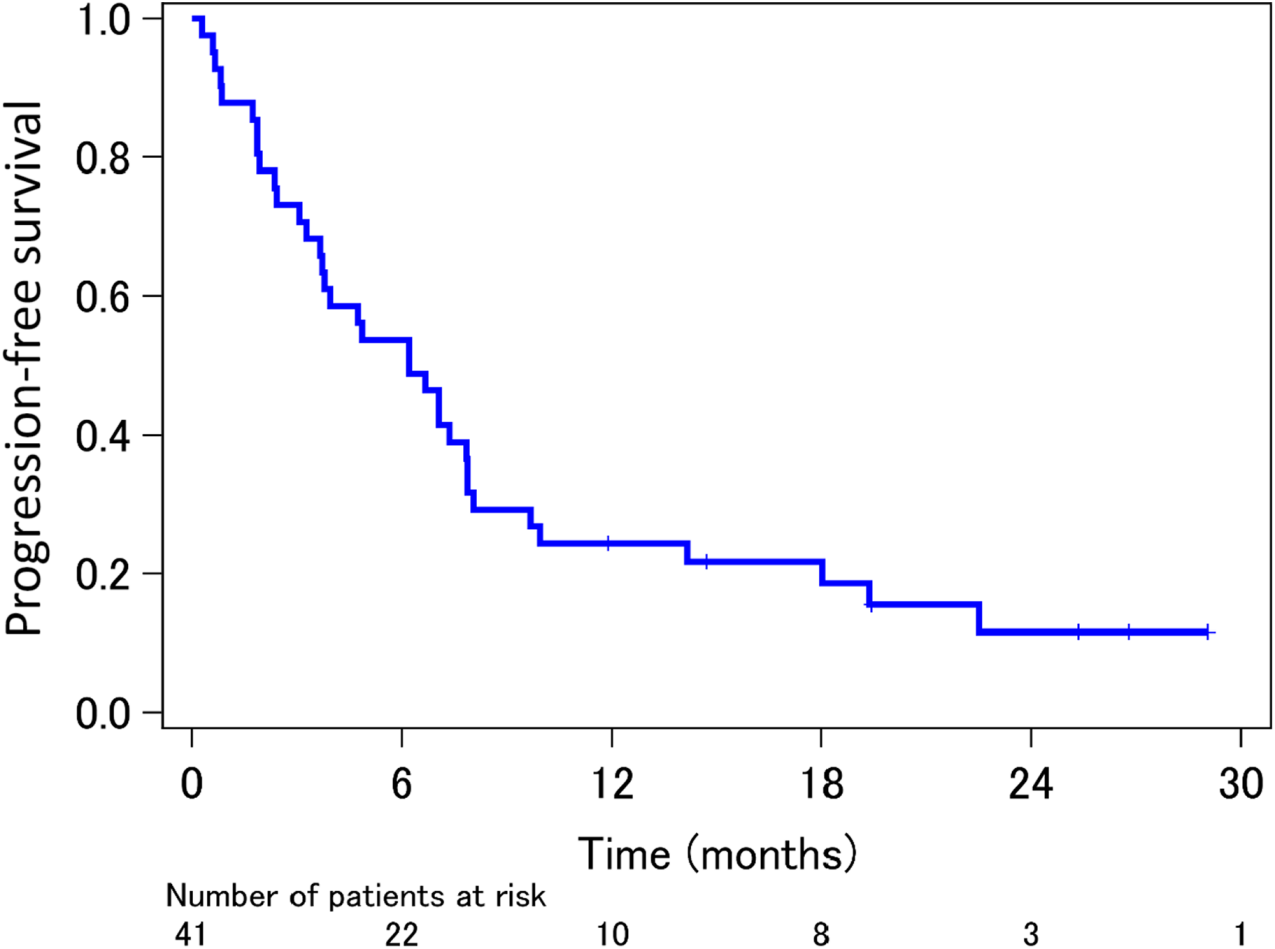
Progression‐free survival after enrollment in the study. The median progression‐free survival time was 6.8 months and median follow‐up time was 6.2 months.

**FIGURE 3:**
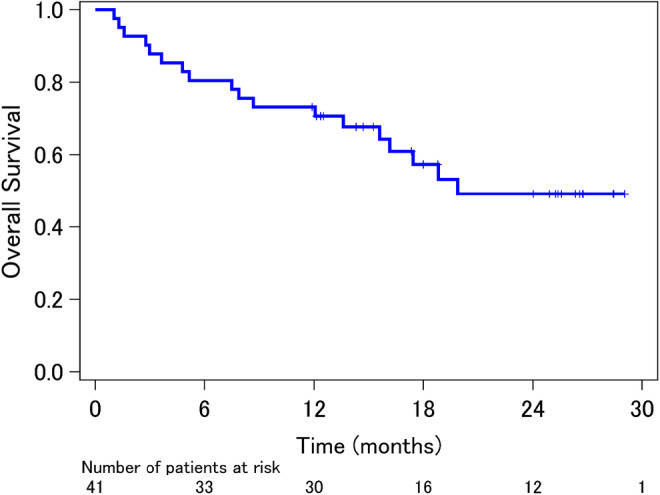
Overall survival after enrollment in the study. The median overall survival time was 21.6 months and median follow‐up time was 15.3 months.

The median PFS was 6.8 months (95% CI, 4.0–8.6 months) and median OS was 21.6 months (95% CI, 17.0 months to not reached) (Figures 2 and 3). Regarding the best objective response, 15 patients (36.6%) had a partial response, 15 (36.6%) had stable disease, and 11 (26.8%) had progressive disease. Overall, the best response rate was 36.6% (95% CI, 21.8%–51.3%). The EGFR‐TKI‐naïve population (15 patients) had better responses than the EGFR‐TKI‐rechallenge population (26 patients): response rate, 60.0% versus 23.1%; median PFS, 8.5 versus 4.8 months (hazard ratio [HR] 0.541; 95% CI, 0.261–1.122); median OS, not reached versus 19.0 months (HR 0.275; 95% CI, 0.079–0.955). Regarding safety during dacomitinib therapy, eight severe adverse events (Grade ≥3) occurred in association with skin toxicities: acneiform rash (three events), paronychia (two events), erythema (two events), and skin infection (one event) (Table S4). Furthermore, severe non‐hematological adverse events (Grade ≥3) occurred in 17 patients (41.5%). Anorexia was the most frequent (six patients, 14.6%), followed by oral mucositis and fatigue (four patients, 9.8%). Interstitial lung disease occurred in two patients and diarrhea in one patient (Table S5).

## 4 DISCUSSION

This was a prospective interventional study with prophylactic treatment to reduce skin toxicity associated with dacomitinib treatment. A total of 41 patients participated, and skin toxicities of Grade ≥2 were observed in 18/41 patients (43.9%; 90% CI, 31.2%–56.7%) during the initial 8 weeks of treatment. The primary endpoint failed to achieve statistical significance because the upper limit of the 90% CI exceeded the pre‐planned threshold of 46%.

The representative skin toxicities with Grade ≥2 in this study were acneiform rash (26.8%) and paronychia (12.2%), which were less than the rates of 33% and 42% in the overall population of the ARCHER1050 study.^2^ The Japanese subset of the ARCHER1050 study, published after the start of our study, showed remarkably higher prevalence of skin toxicities (Grade ≥2) than that of the overall population, with acneiform rash in 70.0% and paronychia in 90.0% of Japanese patients.^12^ Compared with the skin toxicity rates in the Japanese cohort in ARCHER1050, this prophylactic strategy would reduce skin toxicity.

Cohort I in ARCHER1042, which was used as the basis for the statistical design of this study, consisted mostly of non‐Asians, accounting for 97.3% of the enrolled participants. The enzymatic activity of the main metabolizing enzyme of dacomitinib, CYP2D6, is known to differ across races. Asians have a high frequency (41%) of a reduced function allele, CYP2D6*10, which is rarely present in Caucasians.^13^, ^14^ Large racial differences can mislead the calculation of the preset threshold and cause failure to achieve the primary endpoint,^6^ as skin rash during EGFR‐TKI treatment is known to occur more frequently in Asians than in non‐Asians. Racial differences in the incidence of skin rashes after EGFR‐TKI therapy have been observed with erlotinib with rates of 82% in Asians and 68% in non‐Asians and with afatinib with rates of 45.8% in Asians and 35.9% in non‐Asians for paronychia.^15^, ^16^

Cutaneous side effects induced by EGFR‐TKIs are due to the inhibition of EGFR in basal keratinocytes and hair follicles. Disturbance of terminal keratinocyte differentiation and keratinization of the follicular epithelium results in occlusion of the follicular orifice, causing an acne‐like skin rash.^17^, ^18^ Asian populations, possessing smaller terminal hair follicles compared to Caucasians, are inherently predisposed to the occlusion of follicular apertures,^19^ which can contribute the increase of skin rash. Subsequently, this follicular obstruction precipitates a reduction in sebum production, culminating in the proliferation of acne‐associated bacterial flora, leading to cutaneous eruptions.^20^

Individuals of Asian descent, owing to their comparably thinner stratum corneum,^21^ exhibit an elevated sensitivity to cutaneous irritation relative to Caucasians, thereby manifesting an increased prevalence of dermatological reactions. Moreover, the protracted recovery time from barrier damage is an additional contributory factor for skin disorders manifestation among Asians.^22^ Previously reported risk factors for skin toxicity include age, sex, and smoking history,^23^, ^24^ but no obvious risk factors were identified in this study.

Adherence to both oral and topical prophylaxis medications was good, with perfect compliance in 80% of the patients throughout the 8 weeks. Adherence to treatment is crucial for improving quality of life and obtaining optimal outcomes.^25^, ^26^ Factors that influence medication adherence are complex, consisting of medical, personal, economic, and social factors.^27^, ^28^ Based on the main factors of medication adherence, we provided explanation of prevention methods using the instruction guide, performed outpatient checkups at 2‐week intervals, and evaluated adherence using patient diaries. In addition, participation in a clinical trial can lead to the Hawthorne effect, encouraging medication adherence. Interventional studies of adherence are limited, and the evidence is not sufficient for guiding practice in promoting medication adherence among patients with cancer.^29^ However, side effects are a major contributor to non‐adherence,^30^ and preventive interventions for side effects by health care providers can improve patient care.

Skin problems are a challenge in continuing treatment with EGFR inhibitors; 90% of patients develop skin problems, 76% require treatment interruption, 60% require dose reduction, and 32% require treatment discontinuation.^21^, ^28^ In the present study, 51.2% of patients discontinued treatment and 19.5% required dose reduction due to skin problems. Skin toxicity was reduced throughout the entire period of dacomitinib administration.

Response rates were similar to those of other trials in both patients previously treated with EGFR‐TKI and EGFR‐TKI‐naïve patients.^12^, ^31^, ^32^ Among the EGFR‐TKI‐rechallenge population, compared with that in the TOPGAN2020‐02 study, a retrospective analysis of the efficacy of dacomitinib in EGFR‐TKI rechallenge settings, the median PFS was similar, but the median OS in our study was remarkably higher: median PFS, 4.8 versus. 4.3 months and median OS, 19.0 versus. 10.5 months.^32^ The reduction of skin toxicity and lower rate of discontinuation could increase long‐term treatment adherence in the EGFR‐sensitive patient population. The reduction in toxicity may also lead to the maintenance of the patient's well‐being and post‐dacomitinib treatment. However, EGFR‐TKIs including dacomitinib are effective for NSCLC in the front‐line setting, and guidelines recommend first‐line administration in clinical practice.^33^, ^34^, ^35^

The most established prophylaxis for cutaneous toxicity is oral tetracyclines, which prevent and overcome infections.^7^, ^36^ The mechanism of skin damage is thought to be the disruption of the skin barrier due to inflammatory cell infiltration, followed by abnormal homeostasis and inflammation, which is exacerbated by infection and immune activation of the host.^8^, ^23^, ^37^ Considering the entire process of dermatological toxicity, moisturizers to protect the skin barrier and topical steroids to suppress inflammation and cytokines are also important. Moreover, patient education is important for the comprehensive prevention of skin toxicity and continuation of preventive treatment. The ESMO clinical practice guideline for dermatological toxicities related to anticancer agents was published in 2021, and the importance of preventive strategies became more recognized.^29^

This study has some limitations. First, the study had an open‐label, single‐arm design, which could have introduced bias. We tried to educate the patients uniformly using an instruction guide, and we evaluated medication adherence based on patient diaries. Second, previously treated patients were enrolled. Ideally, medication adherence and skin toxicity evaluation should be limited to EGFR‐TKI‐naive patients. In the analyses of the association between skin toxicities and patients' characteristics, no significant correlation was observed. Third, since adherence was good at 8 weeks in this study, concurrently prophylaxis must continue during ongoing EGFR‐TKI therapy. Further study is awaited about long‐term adherence and toxicity management.

This study prospectively evaluated the efficacy of comprehensive prophylaxis for dacomitinib‐related skin toxicity in the Japanese population. Although no significant differences were observed, the study may have been effective considering the racial differences in skin toxicity. The prevention of skin toxicity and patient education are important for the maintenance of quality of life and continuity of treatment.

## AUTHOR CONTRIBUTIONS

**Masahiro Iwasaku:** Data curation (equal); investigation (equal); methodology (equal); resources (equal); software (equal); writing – original draft (equal); writing – review and editing (equal). **Junji Uchino:** Conceptualization (lead); data curation (equal); formal analysis (equal); funding acquisition (lead); investigation (equal); methodology (lead); project administration (lead); resources (equal); writing – original draft (lead); writing – review and editing (equal). **Kenji Chibana:** Data curation (equal); investigation (equal); resources (equal); writing – original draft (supporting); writing – review and editing (supporting). **Shigeru Tanzawa:** Data curation (equal); investigation (equal); resources (equal); writing – original draft (supporting); writing – review and editing (supporting). **Takahiro Yamada:** Data curation (equal); investigation (equal); resources (equal); writing – original draft (supporting); writing – review and editing (supporting). **Kazunori Tobino:** Data curation (equal); investigation (equal); resources (equal); writing – original draft (supporting); writing – review and editing (supporting). **Yasuki Uchida:** Data curation (equal); investigation (equal); resources (equal); writing – original draft (supporting); writing – review and editing (supporting). **Takashi Kijima:** Data curation (equal); investigation (equal); resources (equal); writing – original draft (supporting); writing – review and editing (supporting). **Katsumi Nakatomi:** Data curation (equal); investigation (equal); resources (equal); writing – original draft (supporting); writing – review and editing (supporting). **Miiru Izumi:** Data curation (equal); investigation (equal); resources (equal); writing – original draft (supporting); writing – review and editing (supporting). **Nobuyo Tamiya:** Data curation (equal); investigation (equal); resources (equal); writing – original draft (supporting); writing – review and editing (supporting). **Hideharu Kimura:** Data curation (equal); investigation (equal); resources (equal); writing – original draft (supporting); writing – review and editing (supporting). **Masaki Fujita:** Data curation (equal); investigation (equal); resources (equal); writing – original draft (supporting); writing – review and editing (supporting). **Ryoichi Honda:** Data curation (equal); investigation (equal); resources (equal); writing – original draft (supporting); writing – review and editing (supporting). **Chieko Takumi:** Data curation (equal); investigation (equal); resources (equal); writing – original draft (supporting); writing – review and editing (supporting). **Tadaaki Yamada:** Data curation (equal); investigation (equal); resources (equal); writing – original draft (supporting); writing – review and editing (supporting). **Yoshiko Kaneko:** Data curation (equal); investigation (equal); resources (equal); writing – original draft (supporting); writing – review and editing (supporting). **Fumiaki Kiyomi:** Formal analysis (lead); software (lead); writing – original draft (supporting); writing – review and editing (supporting). **Koichi Takayama:** Conceptualization (equal); funding acquisition (equal); project administration (lead); supervision (lead); writing – original draft (supporting); writing – review and editing (supporting).

## FUNDING INFORMATION

This research was funded by Pfizer. Inc.

## CONFLICT OF INTEREST STATEMENT

Shigeru Tanzawa recieved personal fees as honoraria from Astrazeneca K.K, Lily Japan, Chugai Pharmaceutical and Taiho Pharmaceutical. Tadaaki Yamada recieved commercial research grants from Pfizer, Ono Pharmaceutical, Janssen Pharmaceutical K.K, AstraZeneca, and Takeda Pharmaceutical Company Limited, payment for lectures from Eli Lilly and participated on advisory Board. Koichi Takayama recieved lecture fee from Pfizer Inc.

## ETHICAL APPROVAL

This study was conducted in accordance with the principles of the Declaration of Helsinki. The study protocol and all amendments were reviewed and approved by the ethical review board of the Clinical Research Network Fukuoka Certified Review Board (CRB7180004). All patients provided written informed consent before enrollment.

## CLINICAL TRIAL REGISTRATION

The study protocol has been previously reported and registered in the Japan Registry of Clinical Trials (jRCTs071190015).

## Supporting information


Figure S1.

Table S1.

## Data Availability

The datasets analyzed during the current study are available from the corresponding author on reasonable request.
