# Supplementary material for: Prophylactic treatment of dacomitinib‐induced skin toxicities in epidermal growth factor receptor‐mutated non–small‐cell lung cancer: A multicenter, Phase II trial
Source: Cancer Med. 2023 Jun 3;12(14):15117–27. doi: 10.1002/cam4.6184 (PMC10417098; doi:10.1002/cam4.6184)
Supplement: Supplementary file 1 — Figure S1. [file CAM4-12-15117-s002.pptx]

## Slide 1
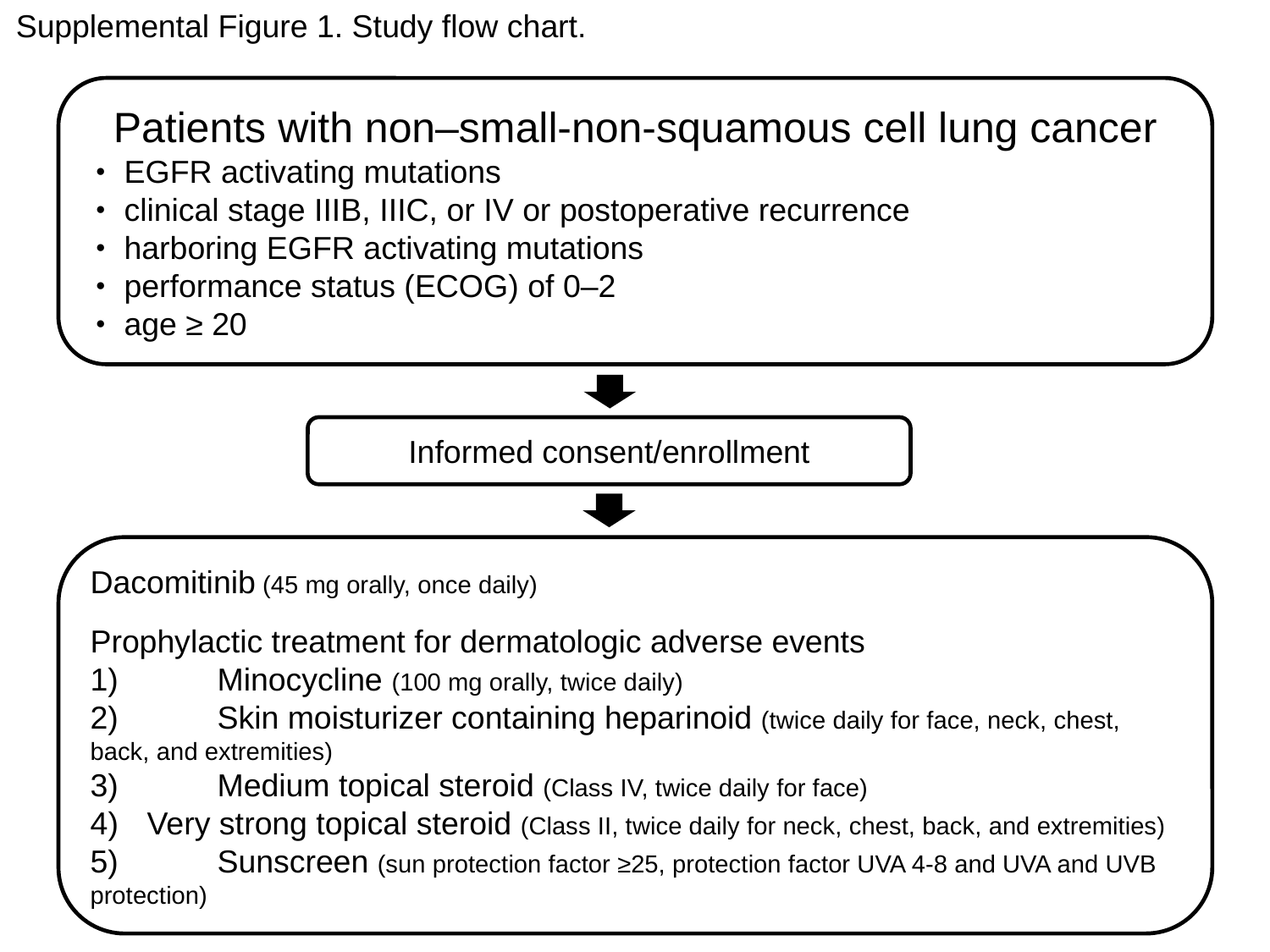

Supplemental Figure 1. Study flow chart.
Patients with non–small-non-squamous cell lung cancer
・EGFR activating mutations
・clinical stage IIIB, IIIC, or IV or postoperative recurrence
・harboring EGFR activating mutations
・performance status (ECOG) of 0–2
・age ≥ 20
Informed consent/enrollment
Dacomitinib (45 mg orally, once daily)
Prophylactic treatment for dermatologic adverse events
1)	Minocycline (100 mg orally, twice daily)
2)	Skin moisturizer containing heparinoid (twice daily for face, neck, chest, back, and extremities)
3)	Medium topical steroid (Class IV, twice daily for face)
 Very strong topical steroid (Class II, twice daily for neck, chest, back, and extremities)
5)	Sunscreen (sun protection factor ≥25, protection factor UVA 4-8 and UVA and UVB protection)
